# Supplementary material for: Benchmarking of computational error-correction methods for next-generation sequencing data
Source: Genome Biol. 2020 Mar 17;21:71. doi: 10.1186/s13059-020-01988-3 (PMC7079412; doi:10.1186/s13059-020-01988-3)
Supplement: Supplementary file 2 — Additional file 2. Review history. [file 13059_2020_1988_MOESM2_ESM.docx]

Review History

**First round of review**

**Reviewer 1**

**Are you able to assess all statistics in the manuscript, including the appropriateness of statistical tests used?**

There are no statistics in the manuscript.

**Comments to author:**

Mitchell et al. implemented a number of error-correction algorithms on short-read next generation sequencing data, using both simulated and real NGS datasets to benchmark methodology performances. They investigated NGS data on different biological backgrounds, with increasing degree of complexity, including whole genome sequencing, immune repertoire sequencing and viral genome sequencing data. While the overall purpose of the manuscript is justified, I find the contribution of this work is only incremental, if any, compared to what previous papers have already conducted, and the conclusions are minimally instructive to the practice of NGS data analysis.

1. Throughout the manuscript, the authors made many comparisons of the methods, and found many inconsistent observations. For example, the heterogeneity of viral genomes is expected to be between WGS and immunogenomics, why the error-correction (EC) algorithms do not work? No explanation was provided, and there was no follow up analysis to figure this out. It seems that genome heterogeneity is not a main confounder for EC algorithms, otherwise BCR repertoire data, which has the highest complexity due to somatic hypermuations, won't have the best results. Another example is, the author mentioned that for BCR-seq data, the performance of Pollux is the worse at read length of 75bp, where 50bp or 100bp both have better gains. Why something in between is the worst? There is no explanation for this observation. In addition, I found it hard to understand why k-mer size sometimes is associated with increased gain, while other times associated with decreased gain. Is this because different datasets have different ways of PCR amplification, rendering different distributions of errors? For a benchmark study comparing different methods, it is the authors' job to inform the reader why certain algorithms do not work as expected. These inconsistencies, and their lack of explanations, weakened the credibility of this work.

2. The authors claimed that EC methods are useful to correct immunogenomics data, but UMI is very commonly applied to most TCR/BCR profiling platforms to reduce sequencing error. The authors assumed UMI may serve as gold standard, so I don't follow the logic why EC methods are necessary here.

3. What's the difference between D2 and D3, or D3 and D4? The D3 dataset was never mentioned in the result section. Also, why using TCR alpha chain to perform the benchmark, where the majority of the TCR repertoire sequencing data is beta chain?

4. It has been reported that for WGS data, regions with lower complexity, or short tandem repeats are more likely to yield inconsistent error correction results. When investigating the sensitivity/precision, the authors could potentially check if the FP or FN calls are due to the presence of these regions.

5. It seems to me that the only actionable suggestion the authors made is to apply EC methods on WGS data with ≥8x coverage. However, many details remain to be explored to make this suggestion more useful, and less incremental (people know that errors should be corrected, and most recently generated WGS data has ≥8 coverage now). For example, which algorithm is mostly recommended? At what read length? Does species matter, or just for human? Does the choice of aligner matter? Should low complexity regions be removed prior to error corrections? And even further: does the application of EC methods improve mutation calling? The current analysis is too thin to draw any meaningful conclusion.

6.  Grammatically mistakes and inaccurate usage of phrases are throughout the manuscript, which really prevented the communication of the scientific message. Half the manuscript was written in present tense, while the other half in past tense. There are many mistakes in the sentences -- For example, the definition of gain metric is written as: 'Following the previous benchmarking? study, we used the gain metric defined as the percentage of sequencing errors errors correctly fixed by the error correction tool ( Table 3 ).' Disregarding the many typos (and a question mark) in this sentence, this definition does not match the mathematical formula in Table 3. This is clearly not a percentage, because it can be negative.

7. (minor point) Figure 4a, should the x and y labels be switched?

8. (minor point) The title mentioned UMI, but this is only part of the test dataset. There is no method developed based on UMI in this method to improve EC methods.

9 (minor) Color of the Supplementary figures represents different algorithms for each figure, making it hard to follow.

**Reviewer 2**

**Are you able to assess all statistics in the manuscript, including the appropriateness of statistical tests used?**

Yes, and I have assessed the statistics in my report.

**Comments to author:**

The NGS-based technologies are indispensable in numerous biology and clinical applications nowadays, while the errors occur in the sequenced data however, is jeopardizing the downstream analysis and limiting the applicability of sequencing technologies in clinical situations to some extent from day one. The computational approaches for error-correction hold promises to eliminate such errors. Yet, how to fairly evaluate the effectiveness of such methods remains problematic. In this work, the authors proposed approaches in evaluating the effectiveness of error-correction algorithms in fixing errors across heterogeneous datasets. The authors first discussed and highlighted the pros and cons of computational error correction approaches in biological fields such as immune-genomics and virology. Then the efficacy of the proposed approach was demonstrated with the adoption of a UMI-based high-fidelity sequencing protocol in eliminating sequencing errors from both simulated data and the real raw data. Then the authors performed a realistic evaluation of error correction methods. The main discovery is that method performance varies substantially across different types of datasets, with no single method performing best on all types of examined data. Finally, the authors offered a good strategy in balancing precision and sensitivity.

Correcting the sequencing error is a very important task and authors' input in this field is contributory and could potentially benefit biological and clinical applications. Enough error-correction methods are included for comparison. Here are a few issues that the authors are suggested to address before being published:

1.     The biochemical and mechanical nature of how errors are generated during sequencing for different sequencing technologies need to be discussed more, as they are highly related to how errors are distributed and affect how error-correction should be developed.
2.     When discussing the distinguishing of error from somatic SNV or germline SNP, the distribution of SNV/SNP in genome space need to be considered and discussed. For example, in cancer genome, the occurrence of SNV is somehow shaped by its 3D genome environment (refer to https://www.nature.com/articles/srep35270); in evolution genome research, the co-occurrence of genome materials is also affected by the chromatin 3D context (refer to https://www.ncbi.nlm.nih.gov/pmc/articles/PMC5416853/).
3.     The author mentioned in Discussion that none of the error-correction methods were able to accurately correct errors in the data derived from the heterogeneous viral populations. Any suggestion how this can be improved? For example, what prior information might be helpful?

**Authors Response**

**Point-by-point responses to the reviewers’ comments:**

Reviewer 1 Remarks to the Author:

Mitchell et al. implemented a number of error-correction algorithms on short-read next generation sequencing data, using both simulated and real NGS datasets to benchmark methodology performances. They investigated NGS data on different biological backgrounds, with increasing degree of complexity, including whole genome sequencing, immune repertoire sequencing and viral genome sequencing data. While the overall purpose of the manuscript is justified, I find the contribution of this work is only incremental, if any, compared to what previous papers have already conducted, and the conclusions are minimally instructive to the practice of NGS data analysis.

Reviewer 1 Specific Comment #1:

Throughout the manuscript, the authors made many comparisons of the methods, and found many inconsistent observations. For example, the heterogeneity of viral genomes is expected to be between WGS and immunogenomics, why the error-correction (EC) algorithms do not work? No explanation was provided, and there was no follow up analysis to figure this out. It seems that genome heterogeneity is not a main confounder for EC algorithms, otherwise BCR repertoire data, which has the highest complexity due to somatic hypermuations, won't have the best results.

*Author Response:*

*We thank the reviewer for raising this important point. We include in our revised manuscript a new dataset, and we performed additional analysis to investigate the factors contributing to the performance of error correction tools on the viral sequencing data. We added the following text to the manuscript:*

*“In contrast with the results produced from immune repertoire sequencing data, none of the error correction tools are suitable for correcting reads derived from the heterogeneous viral populations (D4 dataset). Despite the ability of the methods to correct the majority of sequencing errors, the high number of incorrect adjustments resulted in a decreased precision across all the methods (Figure S18). We performed additional analysis to investigate the factors contributing to the reduced performance of error correction tools on the viral sequencing data. We used a real HIV-1 sequencing benchmark1 composed of five HIV-1 subtype B haplotypes mixed in-vitro (D5 dataset) (Table 1). To prepare error-free reads we have applied haplotype-based error correction protocol able to eliminate sequencing errors by matching the read with the haplotype of origin. After the haplotype and reads were matched, the sequencing errors are corrected by replacing bases from reads with the based from the haplotype of origin. Details about the D5 dataset and haplotype-based error correction protocol are provided in the Supplementary Materials.*

*Similar to results generated from the D4 dataset, the majority of error correction methods were unable to accurately correct errors (Figure S19). Notably, only Racer and Fiona were able achieve gain above 60%; other methods have zero or negative gain. Sensitivity was below 45% for the majority of the methods, except Racer was able to achieve 90.0% sensitivity. Precision ranged from 20% to 99% (Figure S20). The improved performance of error correction tools on D5 HIV mixture dataset can be*

1

*attributed to a small number of haplotypes present in mixture compared to number of haplotypes in the real HIV sample (D4 dataset).*

*We further investigated the factors which can influence the accuracy of error correction in viral sequencing data. First, we varied the diversity between haplotypes. We have generated three datasets each consisting of two haplotypes. The diversity was measured using the Hamming distance and varied between 5.94% and 0.02%. The reduced diversity between haplotypes had a positive effect for majority of error correction method, allowing seven out of 10 methods to achieve positive gain on low-diversity datasets (Hamming distance between haplotypes <0.29%) (Figure S21).”*

*In the revised manuscript, we explained the potential reasons why the results on viral data are generally worse when compared to TCR-Seq data. To clarify this point, we have added the following text to the Discussion section:*

*“One potential reason is the complexity of the HIV virus, which is comprised of multiple similar strains that can confound the performance of the error-correction algorithms. Every region of each HIV viral strain is unique; the distribution of mutations is relatively even across the viral genome when compared to TCR. In contrast, the diversity of TCR is mainly driven by the differences in CDR3 sequence.”*

*To illustrate this new work, we have added the following figures to the revised manuscript:*

**Figure S19.** Barplot depicting the gain across various error correction methods when applied to D5 HIV mixture dataset.

2

**Figure S20.** Scatter plot depicting the sensitivity (x-axis) and precision (y-axis) of each tool when applied to D5 HIV mixture dataset.

**Figure S21.** Heatmap depicting the gain across D5 HIV mixture dataset (with various rates of diversity between haplotypes).

3

*We also added a new section, called “Error correction methods designed for mixed genomes,” in the Supplementary Materials to describe a novel HIV dataset:*

*“We prepared viral dataset D5 using real sequencing data from NCBI with the accession number SRR961514 prepared by Giallonardo et al.1. This is a MiSeq sequencing experiment on a mixture of five subtype B HIV-1 viruses with different genomes. The original dataset contains 714994 MiSeq 2x250bp reads that we mapped on all five HIV-1 reference genomes. Each read was assigned to the reference with which it has a minimum number of mismatches. Since unmapped reads do not have the best match, we dropped them; as a result, there were 706182 remaining reads. The original error rate in the dataset was 1.44%. We modified these reads as follows: first, we corrected the corresponding portion of errors with a corresponding reference nucleotides to obtain different levels of errors in the datasets (1.44%, 0.33%, 0.0033%, 0.0001%); second, we created datasets with original 1.44% error rate and different number of haplotypes in the mixture (1, 2, 5); third, we created datasets with mixtures of two haplotypes with the original 1.44% error rate but with different levels of diversity between haplotypes (Hamming distance=5.94%, 0.29%, 0.02%).”*

*Given the inconsistent results of error correction tools on simulated BCR-Seq data and the lack of experimental gold standard, we decided to omit the results derived from the BCR-Seq data and focus the benchmarking of error correction tools based on TCR-Seq data for the immunogenomics component of the paper.*

Reviewer 1 Specific Comment #2:

Another example is, the author mentioned that for BCR-seq data, the performance of Pollux is the worse at read length of 75bp, where 50bp or 100bp both have better gains. Why something in between is the worst? There is no explanation for this observation.

*Author Response:*

*Given the inconsistent results of error correction tools on simulated BCR-Seq data and the lack of an experimental gold standard, we decided to omit the results of the BCR-Seq data and focus on the benchmarking of error correction tools based on TCR-Seq data for the immunogenomics component of the paper (See Reviewer 1 Specific Comment #1).*

Reviewer 1 Specific Comment #3:

In addition, I found it hard to understand why k-mer size sometimes is associated with increased gain, while other times associated with decreased gain. Is this because different datasets have different ways of PCR amplification, rendering different distributions of errors? For a benchmark study comparing different methods, it is the authors' job to inform the reader why certain algorithms do not work as expected. These inconsistencies, and their lack of explanations, weakened the credibility of this work.

*Author Response:*

*We thank the reviewer for raising this important point. We have added the following text to the*

*revised manuscript:*

4

*“As we observed with the WGS data, with TCR-Seq data an increase in kmer size improves the gain for some of the tools, while for other tools it has no effect (Figure S4, S12).”*

*“Overall, the increase in kmer size results in a decreased number of corrections for all the tools with regards to WGS data (Figure S5).”*

*Given the inconsistent results of error correction tools on simulated BCR-Seq data and the lack of experimental gold standard, we decided to omit the results of the BCR-Seq data and focus the benchmarking on TCR-Seq data.*

Reviewer 1 Specific Comment #4:

The authors claimed that EC methods are useful to correct immunogenomics data, but UMI is very commonly applied to most TCR/BCR profiling platforms to reduce sequencing error. The authors assumed UMI may serve as gold standard, so I don't follow the logic why EC methods are necessary here.

*Author Response:*

*We thank the reviewer for pointing out that this portion of our manuscript required clarifying. We agree with the reviewer that the UMIs are commonly applied for immunogenomics data in order to correct errors. However, associated computational costs of using UMIs may limit the ability to reach acceptable coverage. In this study, we suggest that computational error correction is a promising alternative to using UMIs. As such, the following text has been added to the Discussion section:*

*“These results suggest that computational error correction tools have potential to replace UMI-based error correction protocols. UMIs are commonly applied to data in immunogenomics studies in order to correct sequencing errors, but the computational cost of using UMIs may have a negative impact on the ability to reach adequate coverage.”*

Reviewer 1 Specific Comment #5:

What's the difference between D2 and D3, or D3 and D4? The D3 dataset was never mentioned in the result section. Also, why using TCR alpha chain to perform the benchmark, where the majority of the TCR repertoire sequencing data is beta chain?

*Author Response:*

*We thank the reviewer for pointing out that we failed to define the datasets used in this study. We have clarified the difference between D2 and D3 datasets, as well as D3 and D4, and we added the following text to the Methods section of the paper (modified text is presented in red):*

*“We used both simulated and experimental gold standard datasets derived from human genomic DNA, human T cell receptor repertoires, and intra-host viral populations. The datasets we used correspond to different levels of heterogeneity. The difficulty of error correction increases as the dataset becomes more heterogeneous. The least*

5

*heterogeneous datasets were derived from human genomic DNA (D1 dataset) (Table 1). The most heterogeneous datasets were derived from the T cell receptor repertoire and from a complex community of closely related viral mutant variants (known as quasispecies).”*

*“We used UMI-based clustering to produce error-free reads for the D2 and D4 datasets. Both the D1 and D4 datasets were produced by computational simulations using a customized version of the tool WgSim2 (Figure S1).”*

*In addition, we selected sequencing data generated from alpha chain of TCR to demonstrate the performance of error correction tools on TCR-Seq data. We expect results of TCRB-Seq would be equivalent.*

Reviewer 1 Specific Comment #6:

It has been reported that for WGS data, regions with lower complexity, or short tandem repeats are more likely to yield inconsistent error correction results. When investigating the sensitivity/precision, the authors could potentially check if the FP or FN calls are due to the presence of these regions.

*Author Response:*

*We thank the reviewer for raising this important point. We have compared the gain in lower complexity regions and outside of those regions. The following text has been added to the revised manuscript:*

*“We have also investigated the performance of the tools in the low complexity regions. Excluding the low complexity regions results in a moderate improvement of accuracy for the majority of the tools. The largest difference in performance between low complexity regions and the rest of the genome was evident in results generated by Racer and Pollux. Notably, the only tool with a negative gain for low complexity regions was Pollux (Figure S10).”*

*Figure S10 was added to the manuscript’s Supplemental Materials:*
(a)

6

(b)

**Figure S10.** Heatmap depicting the gain for low complexity regions (‘low’) and the rest of the genome (‘normal’). (a) WGS human data (D1 dataset) with 16x coverage. (b) WGS human data with 32x coverage.

Reviewer 1 Specific Comment #7:

It seems to me that the only actionable suggestion the authors

made is to apply EC methods on WGS data with ≥8x coverage. However, many details remain to be explored to make this suggestion more useful, and less incremental (people know that errors should be corrected, and most recently generated WGS data has ≥8 coverage now). For example, which algorithm is

7

mostly recommended? At what read length? Does species matter,

or just for human? Does the choice of aligner matter? Should low complexity regions be removed prior to error corrections? And even further: does the application of EC methods improve mutation calling? The current analysis is too thin to draw any meaningful conclusion.

*Author Response:*

*We thank the reviewer for sharing these suggestions, which we agree will strengthen the paper. We performed additional experiments to investigate the questions raised by the reviewer. The following text was added to the revised manuscript:*

*“We have also compared the accuracy of error correction algorithm on E. coli WGS data. The relative performance of error correction methods was similar to the human WGS data. However, the differences in performance between the tools on E. coli data were smaller compared to human data specially for high coverage data (Figure 2d-f,j-l). Notably, many tools are able to maintain excellent performance (gain above 90%) even for coverages as low as 8x. The tool with the best performance for low coverage WGS when applied to both human and E. coli data was Coral, which was able to maintain positive gain even for 1x WGS data for E. coli, and 4x for human data (Figure 2g). Precision of error correction tools on E. coli was generally high even for low coverage data (Figure 2h). Many tools are able to achieve sensitivity above 90% even for 8x coverage (Figure 2i). Similar to human data, majority of the tools are able to maintain a good balance between precision and sensitivity for 32x WGS data (Figure 2f,l).”*

*We added results on E. coli data (panels g-l ) to Figure 2:*

8

**Figure 2.** Correcting errors in whole genome sequencing data (D1 dataset). **(a-f)** WGS human data. **(g-l)** WGS *E. coli* data. **(a)** Heatmap depicting the gain across various coverage settings. Each row corresponds to an error correction tool, and each column corresponds to a dataset with a given coverage. **(b)** Heatmap depicting the precision across various coverage settings. Each row corresponds to an error correction tool, and each column corresponds to a dataset with a given coverage. **(c)** Heatmap depicting the sensitivity across various coverage settings. Each row corresponds to an error correction tool, and each column corresponds to a dataset with a given coverage. **(d)** Scatter plot depicting the number of TP corrections (x-axis) and FP corrections (y-axis) for datasets with 32x coverage. **(e)** Scatter plot depicting the number of FP corrections (x-axis) and FN corrections (y-axis) for datasets with 32x coverage. **(f)** Scatter plot depicting the sensitivity (x-axis) and precision (y-axis) for datasets with 32x coverage.

9

*We also investigated the performance of the tools in the low complexity regions (See Reviewer*

*1 Specific Comment #7). Evaluation of the effects of read alignments is beyond the scope of our manuscript. In order to clarify this point, we added the following text to the revised manuscript:*

*“Our benchmarking study focused on benchmarking computational error correction tools. The evaluation of error correction on downstream analyses has been performed and published elsewhere3 and is beyond the scope of the current study.”*

Reviewer 1 Specific Comment #8:

Grammatically mistakes and inaccurate usage of phrases are throughout the manuscript, which really prevented the communication of the scientific message. Half the manuscript was written in present tense, while the other half in past tense. There are many mistakes in the sentences -- For example, the definition of gain metric is written as: 'Following the previous benchmarking? study, we used the gain metric defined as the percentage of sequencing errors errors correctly fixed by the error correction tool ( Table 3 ).' Disregarding the many typos (and a question mark) in this sentence, this definition does not match the mathematical formula in Table 3. This is clearly not a percentage, because it can be negative.

*Author Response:*

*We thank the reviewer for raising this important point. This statement about gain is now clarified*

*and fixed for miscellaneous errors, as shown below:*

*“We used the gain metric4 to quantify the performance of each error correction tool. Positive gain represent an overall positive effect of the error correction algorithm, whereas a negative gain shows that the tool performed more incorrect actions then correct actions. A gain of 1.0 means the error correction tool made all necessary corrections without any FP alterations (Table 3).”*

*We agree that improving the writing of the manuscript would improve the communication of our work. We sent the manuscript for a professional proofreading service, which produced an improved version of the manuscript. We also adjusted the tense to be past tense, when appropriate, throughout the manuscript.*

Reviewer 1 Specific Comment #9:

(minor point) Figure 4a, should the x and y labels be switched?

*Author Response:*

*We thank the reviewer for noticing these improperly labeled axes. The gain is indeed on the x-axis and the method is on the y-axis, which is now indicated properly in Figure 4A, which is now Figure 3a. The new figure and the revised figure caption are included here:*

10

**Figure 3. Correcting errors in TCR-Seq data (D2 dataset). (a)** Bar plot depicting the gain across various error correction methods when applied to TCR-Seq data. Vertical bars depict the various gains across 10 TCR-Seq samples. **(b)** Scatter plot depicting the number of TP corrections (x-axis) and FP corrections (y-axis). Mean value across 10 samples is reported for each tool. **(c)** Scatter plot depicting the number of TP corrections (x-axis) and FP corrections (y-axis). Each dot represents a mean value reported for each tool when applied across 10 samples. **(d)** Scatter plot depicting the sensitivity (x-axis) and precision (y-axis) of each tool. Mean value across 10 samples is reported for each tool.

Reviewer 1 Specific Comment #10:

(minor point) The title mentioned UMI, but this is only part of the test dataset. There is

no method developed based on UMI in this method to improve EC methods.

*Author Response:*

*We thank the reviewer for raising this important point. The title of the paper has now been changed to “Benchmarking of computational error-correction methods for next-generation sequencing data” in order to clearly communicate that some benchmarking of datasets was performed without UMIs.*

Reviewer 1 Specific Comment #11:

(minor) Color of the Supplementary figures represents different algorithms for each

figure, making it hard to follow.

11

*Author Response:*

*We thank the reviewer for raising this important point. The figures throughout the paper now use a consistent coloring scheme for the tools, such that each tool has one unique color representing its results in the figures throughout the entire paper.*

Reviewer 2 Remarks to the Author:

The NGS-based technologies are indispensable in numerous biology and clinical applications nowadays, while the errors occur in the sequenced data however, is jeopardizing the downstream analysis and limiting the applicability of sequencing technologies in clinical situations to some extent from day one. The computational approaches for error-correction hold promises to eliminate such errors. Yet, how to fairly evaluate the effectiveness of such methods remains problematic. In this work, the authors proposed approaches in evaluating the effectiveness of error-correction algorithms in fixing errors across heterogeneous datasets. The authors first discussed and highlighted the pros and cons of computational error correction approaches in biological fields such as immune-genomics and virology. Then the efficacy of the proposed approach was demonstrated with the adoption of a UMI-based high-fidelity sequencing protocol in eliminating sequencing errors from both simulated data and the real raw data. Then the authors performed a realistic evaluation of error correction methods. The main discovery is that method performance varies substantially across different types of datasets, with no single method performing best on all types of examined data. Finally, the authors offered a good strategy in balancing precision and sensitivity.

Correcting the sequencing error is a very important task and authors' input in this field is contributory and could potentially benefit biological and clinical applications. Enough error-correction methods are included for comparison. Here are a few issues that the authors are suggested to address before being published:

Reviewer 2 Specific Comment #1:

The biochemical and mechanical nature of how errors are generated during sequencing

for different sequencing technologies need to be discussed more, as they are highly related to how errors are distributed and affect how error-correction should be developed.

*Author Response:*

*We thank the reviewer for raising this important point. The following text has been added to the*

*Introduction section:*

*“Sequencing errors typically occur in approximately 0.1-1% of bases sequenced; such errors are more common in reads with poor-quality bases where sequencers misinterpret the signal or when the wrong nucleotide is incorporated. Errors are introduced at the sequencing step via incorporation of faults and even occur in reads with few poor-quality bases per read5. Additional errors, such as polymerase bias and incorporation errors, may be introduced during sample preparation, amplification, or library preparation stages6. Data containing sequencing errors limit the applicability of sequencing technologies in clinical settings7. Further, the error rates vary across*

**12**

*platforms8; the most popular Illumina-based protocols can produce approximately one error in every one thousand nucleotides9.”*

*We performed additional experiments to investigate the effect that the number of errors present in the data may have on the ability of benchmarked methods to accurately correct errors. The following text has been added to the revised manuscript:*

*“We have also performed additional experiments to investigate the effect of number of errors present in the data on the ability of methods to accurately correct errors. We have computationally changed the error rate of viral dataset D5 (Supplementary Methods). In total, we have obtained eighth dataset with the error rate ranging from 10-6 to 3.3x10-3. In general, increased error rate had a negative impact on the ability of the majority of the methods to accurate correct errors. Tools, which maintained consistent performance across dataset with various error rates were Fiona and Racer. Notably, Racer was able to maintain gain above 70% across all datasets with various error-rates (Figure S20).”*

*In order to illustrate this point, we also added the following figure to the manuscript:*

**Figure S22.** Heatmap depicting the gain across **D5 HIV mixture dataset (**with various error rates**).**

Reviewer 2 Specific Comment #2:

When discussing the distinguishing of error from somatic SNV or germline SNP, the

distribution of SNV/SNP in genome space need to be considered and discussed. For example, in cancer genome, the occurrence of SNV is somehow shaped by its 3D genome

**13**

environment (refer to [https://www.nature.com/articles/srep35270](https://www.nature.com/articles/srep35270);)); in evolution genome research, the co-occurrence of genome materials is also affected by the chromatin 3D context (refer to [https://www.ncbi.nlm.nih.gov/pmc/articles/PMC5416853/](https://www.ncbi.nlm.nih.gov/pmc/articles/PMC5416853/).)).

*Author Response:*

*We thank the reviewer for raising this important point. We have added the following text to the*

*Discussion section and cite corresponding papers:*

*“In future studies, we anticipate that additional knowledge about the structured*

*properties of analyzed genomes will be used to develop bioinformatics tools that produce more accurate and reliable results. For example, structures of genomes from different organisms are shaped by epistasis resulting in co-dependence of different variants10,11. The incorporation of the effects of epistasis into error-correction methods may help researchers distinguish between real and artificial genomic heterogeneity and eventually result in a higher accuracy of error correction.”*

Reviewer 2 Specific Comment #3:

The author mentioned in Discussion that none of the error-correction methods were able to accurately correct errors in the data derived from the heterogeneous viral populations. Any suggestion how this can be improved? For example, what prior information might be helpful?

*Author Response:*

*We thank the reviewer for raising this important point. We have added the following text to the*

*Discussion section of the revised manuscript (See Reviewer 1 Specific Comment #1):*

*“One potential reason is the complexity of the HIV virus, which is comprised of multiple similar strains that can confound the performance of the error-correction algorithms. Every region of each HIV viral strain is unique; the distribution of mutations is relatively even across the viral genome when compared to TCR. In contrast, the diversity of TCR is mainly driven by the differences in CDR3 sequence.”*

*We also performed additional experiments to investigate the effects of the number of haplotypes and diversity on the performance of error correction tools on the HIV viral data. As such, we added the following text to the Results section of the paper:*

*“We performed additional analysis to investigate the factors contributing to the reduced performance of error correction tools on the viral sequencing data. We used a real HIV-1 sequencing benchmark1 composed of five HIV-1 subtype B haplotypes mixed in-vitro (D5 dataset) (Table 1). To prepare error-free reads we have applied haplotype-based error correction protocol able to eliminate sequencing errors by matching the read with the haplotype of origin. After the haplotype and reads were matched, the sequencing errors are corrected by replacing bases from reads with the based from the haplotype of origin. Details about the D5 dataset and haplotype-based error correction protocol are provided in the Supplementary Materials.*

14

*Similar to results generated from the D4 HIV dataset, the majority of error correction methods were unable to accurately correct errors (Figure S19). Notably, only Racer and Fiona were able achieve gain above 60%; other methods have zero or negative gain. Sensitivity was below 45% for the majority of the methods, except Racer was able to achieve 90.0% sensitivity. Precision ranged from 20% to 99% (Figure S20). The improved performance of error correction tools on D5 HIV mixture dataset can be attributed to a small number of haplotypes present in mixture compared to number of haplotypes in the real HIV sample (D4 dataset).*

*We further investigated the factors which can influence the accuracy of error correction in viral sequencing data. First, we varied the diversity between haplotypes. We have generated three datasets each consisting of two haplotypes. The diversity was measured using the Hamming distance and varied between 5.94% and 0.02%. The reduced diversity between haplotypes had a positive effect for majority of error correction method, allowing seven out of 10 methods to achieve positive gain on low-diversity datasets (Hamming distance between haplotypes <0.29%) (Figure S21).”*

*We include the following updated figures:*

**Figure S19.** Barplot depicting the gain across various error correction methods when applied to D5 HIV mixture dataset.

15

**Figure S20.** Scatter plot depicting the sensitivity (x-axis) and precision (y-axis) of each tool when applied to D5 HIV mixture dataset.

**Figure S21.** Heatmap depicting the gain across D5 HIV mixture dataset (with various rates of diversity between haplotypes).

16

*We also added dataset D5 into Table 1 in the Results section. Added text is highlighted in red*: **Table 1.** Overview of the gold standard datasets.

|  | D1 | D2 | D3 | D4 | D5 |
| --- | --- | --- | --- | --- | --- |
| Technology | Whole genome sequencing | T cell receptor sequencing | T cell receptor sequencing | Viral  sequencing | Viral  sequencing |
| Heterogeneity | Low | High | High | High | High |
| Technique | Simulated | UMI-based  error-free  reads | Simulated | UMI-based error-free reads | Haplotype-based error-free reads |
| Number of samples | 100 | 10 | 200 | 1 | 9 |

*We added the following text to the Methods section of the paper (See Reviewer 2 Specific Comment #3):*

*“Error correction methods designed for mixed genomes*

*We prepared viral dataset D5 using real sequencing data from NCBI with the accession number SRR961514 prepared by Giallonardo et al.1. This is a MiSeq sequencing experiment on a mixture of five subtype B HIV-1 viruses with different genomes. The original dataset contains 714994 MiSeq 2x250bp reads that we mapped on all five HIV-1 reference genomes. Each read was assigned to the reference with which it has a minimum number of mismatches. Since unmapped reads do not have the best match, we dropped them; as a result, there were 706182 remaining reads. The original error rate in the dataset was 1.44%. We modified these reads as follows: first, we corrected the corresponding portion of errors with a corresponding reference nucleotides to obtain different levels of errors in the datasets (1.44%, 0.33%, 0.0033%, 0.0001%); second, we created datasets with original 1.44% error rate and different number of haplotypes in the mixture (1, 2, 5); third, we created datasets with mixtures of two haplotypes with the original 1.44% error rate but with different levels of diversity between haplotypes (Hamming distance=5.94%, 0.29%, 0.02%).”*

*In addition to changes requested by the reviewers, we corrected grammatical errors and edited sentence and paragraph structure for improved clarity and flow.*

17

*We also added one additional analysis and figure to the paper:*

*“We have also compared CPU time and the maximum amount of RAM used by each of the tools based on WGS data (Figure S11). Bless, Racer, RECKONER, Lighter, and BFC were the fastest tools and were able to correct the errors in less than two hours for the WGS sample corresponding to chromosome 21 with 8x coverage. Other tools required more than five hours to process the same samples. The tools with lowest memory footprint were Lighter, SGA, and Musket, requiring less than 1GB of RAM to correct the reads in the samples. The tool with the highest memory footprint was Coral, requiring more than 9GB of RAM to correct the errors.”*

(a)

(b)

**Figure S11.** Barplot depicting (a) the CPU time and (b) the maximum amount of RAM across error correction tools.

18

*References Cited*

*Giallonardo, F. D. et al. Full-length haplotype reconstruction to infer the structure of heterogeneous virus populations. Nucleic Acids Res. 42, e115 (2014).*

*lh. lh3/wgsim. GitHub* [*https://github.com/lh3/wgsim.*](https://github.com/lh3/wgsim.)

*Heydari, M., Miclotte, G., Demeester, P., Van de Peer, Y. & Fostier, J. Evaluation of the impact of Illumina error correction tools on de novo genome assembly. BMC Bioinformatics 18, 374 (2017).*

*Yang, X., Chockalingam, S. P. & Aluru, S. A survey of error-correction methods for next-generation sequencing. Brief. Bioinform. 14, 56–66 (2013).*

*Salk, J. J., Schmitt, M. W. & Loeb, L. A. Enhancing the accuracy of next-generation sequencing for detecting rare and subclonal mutations. Nat. Rev. Genet. 19, 269–285 (2018).*

*Ma, X. et al. Analysis of error profiles in deep next-generation sequencing data. Genome Biol. 20, 50 (2019).*

*Strom, S. P. Current practices and guidelines for clinical next-generation sequencing oncology testing. Cancer Biol Med 13, 3–11 (2016).*

*Robasky, K., Lewis, N. E. & Church, G. M. The role of replicates for error mitigation in next-generation sequencing. Nat. Rev. Genet. 15, 56–62 (2014).*

*Ratan, A. et al. Comparison of Sequencing Platforms for Single Nucleotide Variant Calls in a Human Sample. PLoS One 8, e55089 (2013).*

*Diament, A. & Tuller, T. Tracking the evolution of 3D gene organization demonstrates its connection to phenotypic divergence. Nucleic Acids Res. 45, 4330–4343 (2017).*

*Shi, Y. et al. Chromatin accessibility contributes to simultaneous mutations of cancer genes. Sci. Rep. 6, 35270 (2016).*

**Second round of review**

**Reviewer 1**

The authors have adequately addressed most of the concerns. The only one left is their claim that "UMIs are commonly applied to data in immunogenomics studies in order to correct sequencing errors, but the computational cost of using UMIs may have a negative impact on the ability to reach adequate coverage". Sure, but applying error correction methods also adds to the computational burden. Compared to the accuracy increase, most users will still choose UMI. Can the authors quantify how much computational cost is caused by solving UMIs?

**Reviewer 2**

The authors have answered all my questions and concerns. I think this work is good to be published in Genome Biology.

**Authors Response**

**Point-by-point responses to the reviewers’ comments:**

Reviewer 1 Remarks to the Author:

Reviewer Comment #1: The authors have adequately addressed most of the concerns. The only one left is their claim that "UMIs are commonly applied to data in immunogenomics studies in order to correct sequencing errors, but the computational cost of using UMIs may have a negative impact on the ability to reach adequate coverage". Sure, but applying error correction methods also adds to the computational burden. Compared to the accuracy increase, most users will still choose UMI. Can the authors quantify how much computational cost is caused by solving UMIs?

*Authors’ Response: We thank the reviewer for the comment. We have modified the text in the paper as follows: “UMIs are commonly applied to data in immunogenomics studies in order to correct sequencing errors, but UMI-based error correction may have a negative impact on the coverage and this increases the cost per base of sequencing.”*
